# Coupled mixed-dimensional multiphase porous media approach for modeling airflow, blood flow, and gas exchange in the human lungs

**DOI:** 10.1007/s10237-026-02064-8

**Published:** 2026-04-29

**Authors:** Lea J. Köglmeier, Barbara Wirthl, Carolin M. Eichinger, Buğrahan Z. Temür, Wolfgang A. Wall

**Affiliations:** Institute for Computational Mechanics, Technical University of Munich, Garching bei München, Germany

**Keywords:** Lung modeling, Respiratory mechanics, Perfusion, Gas exchange, Porous media, 0D-3D coupling

## Abstract

Mechanical ventilation is a life-saving therapeutic intervention for patients with impaired pulmonary function, yet it carries the risk of ventilator-induced lung injury (VILI). At bedside, physicians face the challenge of keeping lung tissue in a healthy state while ensuring sufficient gas exchange. Gas exchange occurs between the air in the alveoli and the dense network of pulmonary blood vessels in their walls, and it strongly depends on the balance between ventilation and perfusion. Mismatches between them are a major cause of impaired gas exchange in pulmonary diseases. However, the precise effects of ventilation, including tissue straining on the pulmonary circulation and the connected gas exchange, are largely unknown. Here, we therefore present an approach to computationally model the respiratory and circulatory systems of the human lungs, including gas exchange. Motivated by the lung’s hierarchical structure, our model represents larger airways and blood vessels as spatially resolved discrete networks of zero-dimensional (0D) models that are embedded into a multiphase porous medium (3D). The porous medium models the smaller respiratory and vascular structures, including lung tissue mechanics, in a homogenized way. Additionally, the respiratory gases—oxygen and carbon dioxide—are incorporated as chemical subcomponents of air and blood, with an exchange model in the porous domain. To connect the homogenized (porous domain) and the discrete (networks) representations of airways and blood vessels, we use a 0D-3D coupling method that allows a non-matching spatial discretization of both domains. This comprehensive coupled approach is physics-based, i.e., based on the underlying physical mechanisms, allowing us to investigate the (often unknown and unmeasurable) interplay between ventilation, tissue deformation, perfusion, and its effects on gas exchange dynamics. We anticipate our approach to be an important milestone towards better addressing clinically relevant questions in respiratory care *in silico*, which will contribute to developing improved ventilation strategies and better patient outcomes.

## Introduction

Respiratory diseases are a leading cause of death worldwide, and recently COVID-19 has made the situation even worse: in 2021, this disease alone caused 8.8 million deaths, making it the world’s second biggest killer that year (World Health Organization 2024). Severe cases of respiratory diseases require treatment with invasive mechanical ventilation to ensure the lungs’ main function—the adequate supply of oxygen and the release of carbon dioxide (West and Luks 2016). This undoubtedly vital therapy, however, can also damage the lungs, referred to as ventilator-induced lung injury (VILI). The damage is characterized by inflammatory-cell infiltrates, hyaline membranes, increased vascular permeability, and pulmonary edema (Slutsky and Ranieri 2013), thus, impairing the vital gas exchange interface. The therapy itself can, therefore, be responsible for a deterioration of the patient’s condition and even increase mortality (Nieman et al. 2015). One of the main obstacles in developing more protective ventilation strategies is the still insufficient understanding of the complex mechanical behavior of the lungs—-both in healthy and diseased states. The main reason for this is the lack of insight due to the limited ability for *in vivo* measurements or images at the relevant alveolar level. The precise effects of how ventilation affects the lung microstructure and gas exchange dynamics are largely unknown.

To shed light into this issue (even non-invasively), physics-based computational modeling offers a powerful approach. By building on first principles (in contrast to purely phenomenological methods), it is possible to simulate the underlying mechanical mechanisms and thus even analyze otherwise unknown effects of different ventilation settings in a predictive way. However, lung modeling faces the challenge of capturing the complexity of the organ. This complexity arises from its hierarchical architecture, spanning a wide range of relevant length scales—from the centimeter-sized trachea and main pulmonary artery and vein, which guide airflow and blood flow to the lung periphery, down to the micrometer-scale alveoli and capillaries where gas exchange occurs. Moreover, the lungs comprise multiple interacting physical fields, such as air, blood, tissue and the transport of the respiratory gases oxygen and carbon dioxide.

**Fig. 1:**
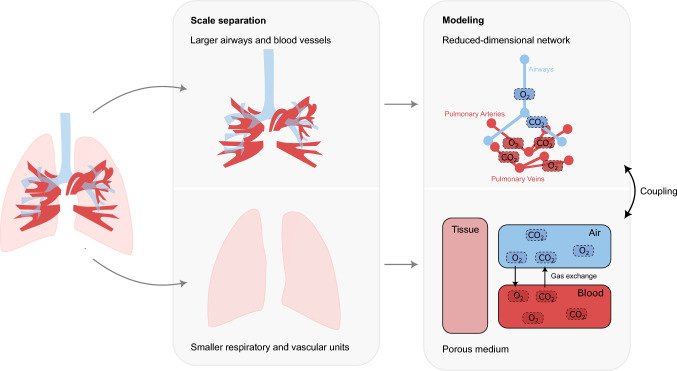
Schematic sketch of the modeling approach, with a scale separation distinguishing between the larger airways and blood vessels and the peripheral smaller respiratory and vascular units in the lung parenchyma. The larger airways and blood vessels are modeled as discrete zero-dimensional networks with the transported respiratory gases oxygen and carbon dioxide (shown on the top right), and the smaller respiratory and vascular units are modeled through a porous medium. The phases (tissue, air, and blood) and species ($O_2$ and $CO_2$) present in the porous domain are outlined on the bottom right

Most of the existing computational models of the lungs focus primarily on the respiratory system, i.e., the interaction between air and lung tissue. The approaches vary in scale and complexity, depending on the problem they aim to address—ranging from computationally efficient reduced-dimensional models of the airways and respiratory zone, for example, in Salmon et al. (1981), Sundaresan et al. (2009), Bates and Irvin (2002), Swan et al. (2012), Ismail et al. (2013), Roth et al. (2017), Geitner et al. (2024), Ryans et al. (2016), to fully resolved three-dimensional simulations of the first airway generations, for example, in Ma and Lutchen (2006); Wall and Rabczuk (2008); Yoshihara et al. (2017), and to representations of the lung parenchyma in a consistent continuum mechanical framework, as in Yoshihara et al. (2017), Berger et al. (2016), Patte et al. (2022), Laville et al. (2023), Peyraut and Genet (2024), Avilés-Rojas and Hurtado (2022), Avilés-Rojas and Hurtado (2025), Badrou et al. (2025). While these models give valuable insights into regional aeration and/or ventilation-induced tissue strain/stress, the coupling with the pulmonary circulation remains largely neglected. "Optimal" ventilation in this sense, however, does not necessarily result in adequate gas exchange, as if "optimal" would just mean minimizing stress and strain, the best ventilation would be no ventilation at all. Effective gas exchange requires a close match between ventilation and perfusion across different lung regions. This becomes evident considering that gas exchange occurs at the alveolar–capillary interface, where a dense network of pulmonary blood vessels is embedded in the alveolar walls. In fact, mismatches between air and blood flow are responsible for most of the impaired gas exchange in pulmonary diseases (West and Luks 2016; Petersson and Glenny 2014; Slobod et al. 2022). In the case of COVID-19, for example, it is suspected that severe hypoxemia in patients with compliant lungs may be due to disrupted perfusion regulation and impaired hypoxic vasoconstriction (Gattinoni et al. 2020). Thus, when studying optimized ventilation, it is crucial to also consider its influence on the blood circulation and the connected, relevant gas exchange. Clark et al. (2011), Burrowes et al. (2011), Roth et al. (2017), Burrowes et al. (2017), Dimbath et al. (2024) have already shown first approaches to couple the respiratory and circulatory system of the lungs. However, all of these approaches still suffer from some shortcomings that limit their usefulness towards reducing VILI. Some of those are for example: The used methods for coupling are often loose, with perfusion calculated from ventilation or tissue mechanics without feedback (Burrowes et al. 2011, 2017). In reality, however, the respiratory and circulatory systems are tightly interconnected: local tissue inflation influences blood vessel patency, whereas increased blood volume can alter tissue distribution profiles and, consequently, regional ventilation (Clark et al. 2017). Moreover, most of the existing models use reduced-dimensional representations of the respiratory zone and are thus limited in their ability to capture inter-alveolar connectivity. As a result, they may fail to capture critical interactions between aerated and collapsed regions—-interactions believed to be central to the development of VILI (Slutsky and Ranieri 2013). Also, most of these models only consider the transport of oxygen, while carbon dioxide is neglected (Roth et al. 2017; Burrowes et al. 2011), despite the fact that its removal plays an important role in clinical practice (Cappadona et al. 2023; Nin et al. 2017).

To overcome these limitations, we present a physics-based computational model of the human lungs that bidirectionally couples the respiratory and circulatory system and, thus, maps the essential feedback loop between ventilation and perfusion, as well as the exchange dynamics of the respiratory gases oxygen and carbon dioxide. Our approach is based on a multiphase porous media formulation that models the smaller respiratory and vascular units in a consistent continuum mechanical framework allowing us to capture complex effects in the respiratory zone like inter-alveolar dependencies. To preserve the hierarchical architecture of the lungs’ major airways and blood vessels, and thus the flow distribution in the lungs, the larger airways and blood vessels are modelled as discrete zero-dimensional networks that are embedded into the deforming porous medium. Further, we model the respiratory gases, oxygen and carbon dioxide, as chemical subcomponents of air and blood with an exchange model in the porous domain. To connect the homogenized and the discrete representations of airways and blood vessels, respectively, a 0D-3D coupling method is used, which allows a non-matching spatial discretization of both domains. We verify the general concept of our approach and show its applicability to patient-specific geometries. Such a comprehensive framework provides a promising base for investigating clinically relevant questions, which will contribute to improved treatment in respiratory care.

The remainder of this paper is structured as follows: in Sect. 2, we present our modeling approach, including the governing equations in the porous medium and the discrete networks, as well as the coupling between both domains. We then present two numerical examples in Sect. 3 to verify our modeling concept and show its applicability to patient-specific geometries. We discuss the results in Sect. 4 and draw a conclusion in Sect. 5.

## Methods

The human lungs exhibit a hierarchical architecture, wherein airflow and blood flow are directed through branching networks of progressively smaller airways and blood vessels to reach the respiratory zone—comprising over 300 million alveoli traversed by dense capillary networks—to enable efficient gas exchange. Motivated by this structure, our modeling approach distinguishes between two domains: the peripheral smaller respiratory and vascular units and the supplying larger airways and blood vessels. The smaller respiratory and vascular units are modeled in a homogenized manner by a multiphase porous medium (described in Sect. 2.1), and the larger airways and blood vessels (arteries and veins) are modeled by discrete networks of zero-dimensional elements (described in Sect. 2.2), similar to an approach that we previously developed for modeling blood vessel networks in a tumor growth model (Kremheller et al. 2019). As a classification criterion that determines when airways or blood vessels, starting from the trachea or the main pulmonary artery/vein, are no longer represented as part of the reduced networks but are modeled in the porous medium, the radius or the generation number is used, depending on the specific question of interest and medical conditions.

The primary function of the lungs is gas exchange: supplying oxygen to the body while eliminating carbon dioxide. To model this clinically relevant issue, both domains include a scalar transport or, in the porous domain, an additional exchange model of the respiratory gases: oxygen ($O_2$) and carbon dioxide ($CO_2$). To connect the homogenized and the discrete representations of airways and blood vessels, both domains are coupled by tissue deformations and by the exchange of fluid and respiratory gases at the tips of the discrete networks, which will be described in Sect. 2.3. The entire modeling approach is depicted in Fig. 1.

### Smaller respiratory and vascular units as a multiphase porous medium

**Fig. 2:**
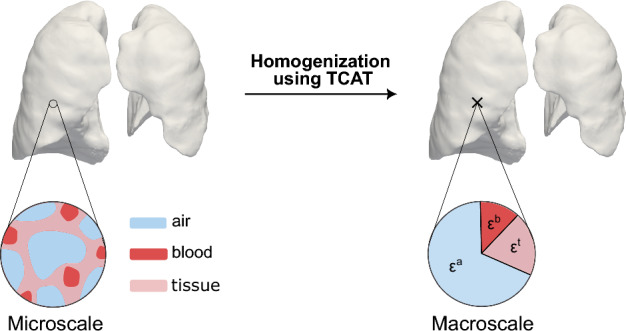
Schematic sketch of the multiphase porous medium modeling the lungs’ smaller airways and blood vessels. At the microscale, the phases and interfaces are discernible (left side), while at the macroscale, the phases are no longer fully resolved but described by their volume fractions, i.e., the volume fraction of air $\varepsilon ^a$, blood $\varepsilon ^b$, and tissue $\varepsilon ^t$ (right side). The thermodynamically constrained averaging theory (TCAT) formally averages the microscale quantities to the macroscale (Miller et al. 2022)

The smaller respiratory and vascular units in the human lungs have a complex structure consisting of small airways ending in over 300 million alveoli and a network of small blood vessels traversing the extremely thin alveolar walls. The patient-specific geometry on the microscale, with fully resolved phases and interfaces (see the left side of Fig. 2), is not available due to the limited resolution of current imaging techniques. It is, however, also not required to answer our questions of interest. We are not interested in the processes in individual alveoli or capillaries but rather in the resulting flows, strains, and stresses on a larger scale, the macroscale. The macroscopic length scale corresponds to the classical definition of a representative elementary volume (REV) (Gray and Miller 2014). This length scale is sufficiently large to capture the averaged microscale conditions, yet small enough to accurately represent the relevant physical effects in the lungs we are interested in.

For modeling the smaller respiratory and vascular units at the macroscale, we use a multiphase porous media formulation based on the thermodynamically constrained averaging theory (TCAT) (Gray and Miller 2014). The TCAT approach formally averages the microscale quantities to the macroscale incorporating thermodynamics to provide a scale-consistent model formulation (Miller et al. 2022). At the macroscale the phases and interfaces are no longer fully resolved, but are modelled by averaged quantities as their volume fractions $\varepsilon$ (see right side in Fig. 2).

In this study, the porous medium consists of three phases, which are one solid phase, i.e., the skeleton of the porous medium representing lung tissue, and two fluid phases—air and blood—flowing through the pores of the skeleton. The pore space is, therefore, divided into two separate porous networks, distinguishing between pores containing air and pores containing blood. We assume a fully saturated porous medium, so the overall porosity of the domain $\varepsilon$ is defined by the sum of the volume fractions of air $\varepsilon ^a$ and blood $\varepsilon ^b$, i.e.,

1 $$\begin{aligned} \varepsilon = \varepsilon ^a + \varepsilon ^b, \end{aligned}$$

resulting in a volume fraction of the tissue of

2 $$\begin{aligned} \varepsilon ^t = 1 - \varepsilon = 1 - \varepsilon ^a - \varepsilon ^b. \end{aligned}$$

For describing the flow and the deformation state in the porous medium, the macroscale conservation of mass and the macroscale conservation of momentum form the basic building blocks. The governing equations based on TCAT are already derived by Gray and Schrefler (2001), Rybak et al. (2015), Jackson et al. (2009), Gray and Miller (2009) and are applied to our system in the following sections. The equations are formulated in a consistent Arbitrary Lagrangian–Eulerian formulation (ALE) (Donea et al. 2004), with the Lagrangian observer following the solid motion as it is classically done in the field of poromechanics.

#### Lung tissue as solid phase

*Conservation of momentum.* The lung tissue is the solid phase of our porous medium $\Omega$, often called the skeleton. It separates the fluid phases air and blood, which are flowing through and interacting with its pores. To consider large deformations of the tissue during breathing, we distinguish between the material (reference) configuration $\Omega _0$, described by the material coordinates $\boldsymbol{X}$, and the spatial (current) configuration $\Omega _t$, described by the spatial coordinates $\boldsymbol{x}$. For the purpose of this study, the end-expiratory state can be taken as the stress-free reference configuration. The governing equation of the tissue is given by the macroscale conservation of momentum (Gray and Miller 2014). For simplicity of this presentation, we assume no body forces acting on the domain and neglect inertia effects. The momentum balance equation of the tissue in spatial configuration is given by

3 $$\begin{aligned} \boldsymbol{\nabla } \cdot \left( \varepsilon ^t \boldsymbol{\sigma }^{t} \right) - \sum _{\kappa \in J_{nt}} \overset{\kappa \rightarrow t}{\boldsymbol{t}_0} = 0 \, \, \, \, \, \, \, \, \text {on} \, \, \Omega _t \times [t_0, t_e], \end{aligned}$$

where $\varepsilon ^{t}$ and $\boldsymbol{\sigma }^{t}$ define the volume fraction and the Cauchy stress tensor of the lung tissue, respectively. Further, $\overset{\kappa \rightarrow t}{\boldsymbol{t}_0}$ represents the interaction forces between adjacent phases $\kappa$, summarized in the set $J_{nt}$. In poromechanics, instead of solving this momentum balance equation for the solid phase in isolation, it is common practice to solve the momentum balance equation for the entire porous system, i.e., the sum of the momentum equations of all present phases, referred to as the mixture. As a result, the interaction forces between phases cancel out, simplifying the representation of the system. The total stress of the mixture is defined as

4 $$\begin{aligned} \boldsymbol{\sigma }^{tot} = \boldsymbol{\sigma }^{eff} - p^t \boldsymbol{I}, \end{aligned}$$

with the identity tensor $\boldsymbol{I}$, the effective stress tensor $\boldsymbol{\sigma }^{eff}$ and the tissue pressure $p^t$ (Coussy 2004; G. Gray and A. Schrefler 2007). The tissue pressure accounts for the stresses exerted from the fluid phases on the skeleton and is commonly defined as the weighted sum of the pressures in the pore space (Kremheller et al. 2018), i.e., the pressure of air $p^a$ and the pressure of blood $p^b$, as

5 $$\begin{aligned} p^t = \frac{\varepsilon ^a}{\varepsilon ^a + \varepsilon ^b} \cdot p^a + \frac{\varepsilon ^b}{\varepsilon ^a + \varepsilon ^b} \cdot p^b. \end{aligned}$$

The effective stress tensor accounts for stress effects due to changes in porosity and the deformation of the tissue (G. Gray and A. Schrefler 2007). With the assumption of a constant tissue density $\rho ^t$, the effective stress can be calculated by the derivative of a strain energy function $\Psi ^t$, as it is done in solid mechanics for hyperelastic materials (Coussy 2004). In the course of this study, we use a simple Neo Hookean material model, which is defined by the strain energy function

6 $$\begin{aligned} \begin{aligned} \Psi _{NH}&= \frac{G}{2} \left( tr \left( \boldsymbol{F}^{T} \boldsymbol{F}\right) - 3\right) \\ &+ \frac{G}{2 \beta } \left( J^{-2 \beta } -1 \right) , \, \, \text {with} \, \, \, \beta = \frac{v}{1-2v}, \end{aligned} \end{aligned}$$

where *G* is the shear modulus, *v* is Poisson’s ratio and $\boldsymbol{F}$ the deformation gradient with its determinant *J* (Holzapfel 2000). The deformation gradient is defined as

7 $$\begin{aligned} \boldsymbol{F} = \frac{\partial \boldsymbol{x}}{\partial \boldsymbol{X}} = \boldsymbol{I} + \frac{\partial \boldsymbol{d}^t}{\partial \boldsymbol{X}}, \end{aligned}$$

with the displacements of the tissue $\boldsymbol{d}^t$. Including more advanced tissue constitutive laws is straightforward within the present framework, should future research questions require it. The final balance of momentum of the mixture with pull back to the material configuration $\Omega _0$ is given by

8 $$\begin{aligned} \begin{aligned} \boldsymbol{\nabla }_0 \cdot \left( \boldsymbol{F} \, \boldsymbol{S}^{tot} \right)&= \boldsymbol{\nabla }_0 \cdot \left( \boldsymbol{F} \, \left( \boldsymbol{S}^{eff} - p^t J \boldsymbol{F}^{-1} \boldsymbol{F}^{-T} \right) \right) \\ &= \boldsymbol{0} \, \, \, \, \, \, \, \, \text {on} \, \, \Omega _0 \times [t_0, t_e], \end{aligned} \end{aligned}$$

with the material divergence operator $\boldsymbol{\nabla }_0 \, \cdot$, the second Piola-Kirchhoff total stress tensor $\boldsymbol{S}^{tot}$ and the second Piola-Kirchhoff effective stress tensor $\boldsymbol{S}^{eff}$. This equation has to be solved for the primary variables: the displacements of the tissue $\boldsymbol{d}^t$.

*Conservation of mass.* The mass conservation equation of the lung tissue enters our system in integral form, as also done in Kremheller et al. (2018). We model the lung tissue as incompressible due to its high water content (and the well-grounded assumption that water can be seen as incompressible in the physiological relevant timescales (Roth et al. 2017)) and assume no mass transfer between the tissue and other system phases. The governing balance of mass of the lung tissue yields

9 $$\begin{aligned} \begin{aligned} \int \limits _{\Omega _0} \rho _0^t \left( 1 - \varepsilon _0^a -\varepsilon _0^b \right) \textrm{d}V_0 = \int \limits _{\Omega _t} \rho _0^t \left( 1 - \varepsilon ^a -\varepsilon ^b \right) \textrm{d}V \\ = \int \limits _{\Omega _0} \rho _0^t \left( 1 - \varepsilon ^a -\varepsilon ^b \right) J \textrm{d}V_0, \end{aligned} \end{aligned}$$

where the subscript 0 indicates the initial state of the quantities. As this equation holds for volumes of arbitrary sizes, we can use the localization theorem and deduce the following relation between the volume fractions

10 $$\begin{aligned} \varepsilon ^a = 1 - \varepsilon ^b - \frac{1 - \varepsilon ^a_0 - \varepsilon ^b_0}{J}. \end{aligned}$$

#### Air and blood as fluid phases

*Conservation of momentum.* Starting from the macroscale conservation of momentum for a generic fluid phase *f*, representing air and blood $f \in \{a,b\}$, in the porous domain, we apply the following assumptions: (1) inertia terms are neglected due to the slow dynamics in the smaller airways and blood vessels during normal breathing (as also done, e.g., in Berger et al. (2016) and Erbertseder et al. (2012)) (2) body forces are neglected for simplicity, and (3) momentum exchange resulting from interface mass transfer is neglected as they are of the same order of magnitude as inertial effects (Sciumè et al. 2013). Further, the macroscopic stress tensor of the fluid phase $\boldsymbol{\sigma }^f$ is commonly defined by its pressure as

11 $$\begin{aligned} \boldsymbol{\sigma }^f = - p^f \boldsymbol{I}. \end{aligned}$$

This results in a conservation of momentum equation of a fluid phase *f* in the porous medium given by the well-known Darcy-equation

12 $$\begin{aligned} \boldsymbol{\nabla } p^f + \varepsilon ^f \mu ^f (\boldsymbol{k}^{f} ) ^{-1} \cdot \left( \boldsymbol{v}^f - \boldsymbol{v}^t \right) = \boldsymbol{0} \, \, \, \, \, \, \, \, \text {on} \, \, \Omega _t \times [t_0, t_e], \end{aligned}$$

with the fluid viscosity $\mu ^f$, the velocity of the fluid $\boldsymbol{v}^f$, the velocity of lung tissue $\boldsymbol{v}^t$, and the permeability tensor ${\boldsymbol{k}^f}$. The permeability tensor depends only on the microscale geometry and is assumed isotropic in the course of this study. It is defined by the constant permeability coefficient $k^f$ and the identity tensor $\boldsymbol{I}$ as

13 $$\begin{aligned} \boldsymbol{k}^f =k^f \cdot \boldsymbol{I}. \end{aligned}$$

##### Remark

In reality, the structure of the smaller airways and blood vessels in the parenchymal region is, in general, not isotropic but follows a hierarchical architecture, where terminal airways and arteries always supply the same geometrically defined regions (West and Luks 2016). To accurately represent this characteristic, more advanced anisotropic permeability models should be considered in future studies.

*Conservation of mass.* The macroscale conservation of mass for a generic fluid phase *f* in the multiphase pore space formulated in the ALE description reads as follows:

14 $$\begin{aligned} \begin{aligned} \frac{\partial \, (\varepsilon ^{f} \rho ^f) }{\partial t} \bigg |_{\boldsymbol{X}}&+ \varepsilon ^{f} \rho ^f \, \boldsymbol{\nabla } \cdot \boldsymbol{v}^t + \boldsymbol{\nabla } \cdot \left( \varepsilon ^{f} \rho ^f \left( \boldsymbol{v}^f - \boldsymbol{v}^t \right) \right) \\ &- \sum _{\kappa \in J_{nf}} \overset{\kappa \rightarrow f}{M}\ = 0 \, \, \, \, \, \, \, \, \text {on} \, \, \Omega _t \times [t_0, t_e], \end{aligned} \end{aligned}$$

with the fluid density $\rho ^f$, the volume fraction of the phase $\varepsilon ^{f}$ and the material coordinate $\boldsymbol{X}$. The last term on the left-hand side describes a sum of possible mass exchanges with a neighboring phase $\kappa$, where $J_{nf}$ denotes the set of all neighboring phases. In the context of this study, no interfacial exchange is considered. So this term is only used for the coupling to the discretely modeled airways and blood vessels, respectively, which will be described in Sect. 2.3.

Due to the neglection of inertial effects (see assumption 1), the Darcy-equation (Eq. 12) can now be directly inserted into Eq. (14) reading as

15 $$\begin{aligned} \begin{aligned} \frac{\partial \left( \varepsilon ^f \rho ^f \, \right) }{\partial t} \bigg |_{\boldsymbol{X}}&+ \varepsilon ^f \rho ^f \, \boldsymbol{\nabla } \cdot \boldsymbol{v}^t - \boldsymbol{\nabla } \cdot \left( \rho ^f \, \frac{\boldsymbol{k}^f}{\mu ^f} \cdot \boldsymbol{\nabla } p^f \right) \\ &- \sum _{\kappa \in J_{nf}} \overset{\kappa \rightarrow f}{M}\ = 0 \, \, \, \, \, \, \, \, \text {on} \, \, \Omega _t \times [t_0, t_e]. \end{aligned} \end{aligned}$$

The velocity vector of the considered fluid phase $\boldsymbol{v}^f$ is thus substituted from the mass conservation equation. This means that the fluid velocity no longer has to be solved as an independent primary variable, but can be calculated based on Eq. (12), as for example also done by Sciumè et al. (2013). Thus, Eq. (15) represents the final equation governing the flow of air and blood, which has to be solved for the primary variable $p^f$.

The presented conservation Eqs. (8), (10), (15) that build the basis of our model contain more unknowns than equations (Rybak et al. 2015). With the introduction of a second fluid phase in the porous medium, we have to apply an additional relationship to close the system of equations. In particular, we need to specify an equation describing the evolution of the volume fraction of blood.

#### Evolution equation for the volume fraction of blood

Because the blood volume fraction changes during breathing, we need to specify an equation describing its evolution. In general, the need for evolution equations for geometric properties is rooted in the fact that these quantities do not exist at the microscale but arise in the averaging process. The evolution equations are independent of system physics as described by conservation and thermodynamic equations, but are based on the system kinematics (Gray and Miller 2014). The derived conservation equations from the previous sections must, therefore, be supplemented by an additional kinematic equation (Gray et al. 2015) describing the evolution of the volume fraction of blood during breathing. The exact kinematic behavior of blood volume during breathing is an area of current research (Ribeiro et al. 2024; Zang et al. 2025), but it is known to be mainly influenced by two phenomena: the deformation of the surrounding tissue and the pressure within the surrounding alveoli (West and Luks 2016).

**Fig. 3:**
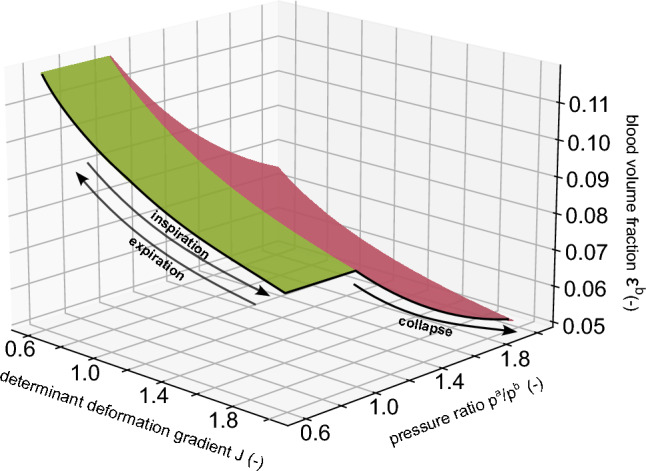
Evolution equation of the volume fraction of blood $\varepsilon ^b$ (see Eqs. 16 and  17). Its behavior depends on the initial volume fraction of blood (here $\varepsilon _0^b = 0.1$), the determinant of the deformation gradient *J*, the pressure of air $p^a$, the pressure of blood ${p^b}$ and the parameters $k_J = -1/3$ and $k_p = -2/3$ determining the deformation dependence (shown in green) and pressure dependence (shown in red)

Considering the first, we distinguish between extra-alveolar and alveolar vessels (West and Luks 2016). Concerning the extra-alveolar vessels, their blood volume is influenced by the deformation of the surrounding tissue that they expand as the lung volume expands during inspiration and shrink as the lung volume decreases during expiration. Concerning the alveolar vessels/units, their blood volume is influenced by the deformation of the surrounding tissue (alveoli) in the opposite way compared to the extra-alveolar vessels: during inspiration, the smaller alveolar vessels in the tissue are compressed as the alveoli expand, leading to a decrease in the volume fraction and vice versa during expiration. To capture this phenomenon, we postulate an evolution equation for the volume fraction of blood $\varepsilon ^b$ in the form

16 $$\begin{aligned} \varepsilon ^b(J) = \varepsilon _0^b \cdot J^{k_J}, \end{aligned}$$

with the initial volume fraction of blood (usually at end-expiration) $\varepsilon _0^b$ and the determinant of the deformation gradient *J*. The parameter $k_J$ determines the deformation dependence. It should be specified based on the number of blood vessel generations modeled within the porous domain. Setting the parameter to $-1$ will reflect that the total blood volume stays constant during breathing. Larger values of $k_J$ increase the total blood volume during inspiration representing the extra-alveolar vessels. Smaller values decrease the total blood volume during inspiration representing the alveolar vessels. Equation (16) is visualized in Fig. 3 by the green color.

Considering the second, the blood volume depends on the pressure of the surrounding alveoli, as they are only separated by thin tissue walls. The most dominant effect is when alveolar pressure exceeds the pulmonary blood pressure, resulting in the capillaries collapsing. This leads to a significant reduction in blood volume referring to West’s zone 1 (West and Luks 2016). This condition, does not occur during healthy states but is highly likely to occur when patients are mechanically ventilated at high pressures (for example, during recruitment maneuvers aiming to open collapsed lung units) and therefore, in the special interest of this study. When using the classification from above in alveolar and extra-alveolar vessels, this effect is predominant in the former and has less influence in the latter.

To additionally capture this effect, we extend the evolution equation of blood as follows:

17 $$\begin{aligned} \varepsilon ^b (J, p^a, p^b) = {\left\{ \begin{array}{ll} \varepsilon ^b(J) \cdot {\left( \frac{p^a}{p^b} \right) }^{k_p} & \text {if}\ \left( \frac{p^a}{p^b} \right) > 1\\ \varepsilon ^b(J) & \text {otherwise} \end{array}\right. } \end{aligned}$$

Here, $p^a$ is the pressure of air, ${p^b}$ is the pressure of blood, and $k_p$ is the parameter determining the pressure dependence, which must be negative to represent the described collapse condition. The magnitude controls the intensity of the collapse and can, therefore, be adjusted according to the area mapped by the porous medium. The behavior according to the final equation is presented in Fig. 3 with the deformation dependence visualized in green and the pressure dependence visualized in red. We set the parameters $k_J = -1/3$ and $k_p = -2/3$ to show a physiological condition in the lungs modeling extra-alveolar and alveolar vessels in the porous domain, where the volume fraction of blood decreases (but the total blood volume increases) as the lungs expands during inspiration, and decrease strongly if the collapse condition is fulfilled. The effect of these newly introduced parameters should clearly be further analyzed, for example using uncertainty quantification methods as proposed, for example, by Wirthl et al. (2023) and Nitzler et al. (2022).

##### Remark

It should be noted that our proposed evolution equation of the blood volume fraction does not directly incorporate the influence of changes in transmural pressure, the pressure difference between air and blood, but only focuses on the most dominant effect, when the pressure of air exceeds the blood pressure. Incorporating the effect of the transmural pressure would increase the complexity of the model and require additional parameters, which are hard to identify. The influence of this simplification should be analyzed in further studies.

#### Oxygen and carbon dioxide as species

This section models the transport and exchange of the respiratory gases: oxygen and carbon dioxide. The gases are chemical subcomponents of the fluid phases air and blood $f \ \epsilon \ \{ a,b\}$ and are modeled as a scalar species $i \ \epsilon \ \{ O_2, CO_2 \}$ defined by their mass fractions $\omega ^{if}$ in the corresponding fluid phase.

##### Remark

In general, following the TCAT approach, the macroscopic conservation equations can be derived for species in a phase and for the phase as a whole. Thus, the latter can be derived either from the averaging of the microscale counterparts, as described in Sects. 2.1.1 and 2.1.2, or by summing up the macroscale conservation equations of all individual species that make up the phase. However, the phases in our model consist of various species whose behavior is not crucial to the question of lung modeling. A complete resolution of the phase into all components would be computationally very expensive without generating useful insights. Therefore, it is common practice, and also done in this study, to solve the conservation equations for all existing phases as a whole in combination with the scalar conservation equation of the species of interest, in this case, oxygen and carbon dioxide.

The governing equation for a species *i* dispersed in a phase *f* is given by the instationary convection-diffusion–reaction equation (Gray and Schrefler 2001; Kremheller et al. 2018; Sciumè et al. 2013), written in ALE-formulation as

18 $$\begin{aligned} \begin{aligned} \varepsilon ^f \rho ^f \, \frac{\partial \omega ^{if}}{\partial t} \bigg |_{\boldsymbol{X}}&- \rho ^f \frac{\boldsymbol{k}^f}{\mu ^f} \nabla p^f \cdot \nabla \omega ^{if} - \nabla \cdot \left( \rho ^f \varepsilon ^f \boldsymbol{D}_{eff}^{if} \nabla \omega ^{if} \right) \\ &+ \omega ^{if} \sum _{\kappa \in J_{nf}} \overset{\kappa \rightarrow f}{M}\ - \sum _{i \kappa \in J_{nif}} \overset{i \kappa \rightarrow if}{M}\ \\ &= 0 \, \, \, \, \, \, \, \, \text {on} \, \, \Omega _t \times [t_0, t_e], \end{aligned} \end{aligned}$$

with a convective velocity given by Darcy’s law and a diffusive contribution based on Fick’s law with the effective diffusion tensor $\boldsymbol{D}_{eff}^{if}$. We assume an isotropic case, defining the effective diffusion tensor by the constant diffusion coefficient $D^{if}$ and the identity tensor $\boldsymbol{I}$ as

19 $$\begin{aligned} \boldsymbol{D}_{eff}^{if} = D^{if} \boldsymbol{I}. \end{aligned}$$

More advanced relationships for the macroscale effective diffusion tensor, e.g., accounting for the phase volume fraction or the tortuosity of the porous domain, as presented in Santagiuliana et al. (2016) and Sciumè et al. (2014), are straightforward to implement, however, not in the focus of this study. The terms on the right side of Eq. (18) describe the mass exchange between adjacent phases (first term) and between species in adjacent phases (second term). With $J_{nf}$ and $J_{nif}$ defining the set of all neighboring phases and species in these phases, respectively. The first is already defined in Eq. (14). The second term is made up of two contributions: the coupling with the respiratory gases in the reduced-dimensionally modeled larger airways and blood vessels, which is presented in Sect. 2.3, and the exchange of oxygen and carbon dioxide between air and blood.

This exchange is driven by the partial pressure difference of the gases between air and blood and takes place by diffusion described by Fick’s law (West and Luks 2016; Klinke et al. 2010) as

20 $$\begin{aligned} \overset{ia \rightarrow ib}{M}\ = \rho ^i \, D_L \cdot \left( p^{ia} - p^{ib} \right) , \end{aligned}$$

with $\rho _i$ being the density of the gas *i*, $D_L$ the diffusion capacity of the lungs, and $p^{ia}$ and $p^{ib}$ the partial pressures of the gas *i* in air and blood, respectively. The diffusion capacity $D_L$ is defined by the diffusion coefficient of the gas in tissue $D^{it}$, the water solubility of the gas $\alpha ^i$, the thickness of the blood-air barrier *d*, and the exchange surface-to-volume relation $\frac{S}{V}$ (Klinke et al. 2010). To account for inhomogeneous blood distributions in the lungs it is further scaled with a ratio of the current volume fraction of blood to its healthy state $\frac{ \varepsilon ^b}{\varepsilon ^{Healthy}}$ given as

21 $$\begin{aligned} D_L = D^{it} \cdot \frac{ \varepsilon ^b}{\varepsilon ^{Healthy}} \cdot \alpha ^i \, \cdot \frac{1}{d} \cdot \frac{S}{V} . \end{aligned}$$

To apply Fick’s law, the partial pressure of the gases in air and blood is required. However, the governing conservation equation for the gases is formulated using the mass fraction of the gases as their primary variable. Therefore, a conversion between partial pressure and mass fraction is necessary.

In air, the partial pressure of oxygen and carbon dioxide is defined by the volume ratio of the gases to the phase (Klinke et al. 2010). By scaling with the densities, we obtain a final relationship between partial pressure and mass fraction of the respiratory gases in air as

22 $$\begin{aligned} \frac{\rho ^i}{\rho ^a} \frac{V^i}{V^a} = \omega ^{ia} = \frac{\rho ^i}{\rho ^a} \frac{ p^{ia}}{p^a}. \end{aligned}$$

In blood, the transport of oxygen and carbon dioxide follows more complex mechanisms, requiring a distinction between the gases in the subsequent analysis.

Starting with oxygen, it is present in blood in two forms: physically dissolved and bound to hemoglobin. Taking this property into account, we use the following relationship between the partial pressure $p^{O_2b}$ and the mass fraction $\omega ^{O_2b}$ of oxygen in the blood, similar to Kremheller et al. (2019) and Possenti et al. (2021):

23 $$\begin{aligned} \begin{aligned} \omega ^{O_2b}(p^{O_2b})&= \frac{\rho ^{O_2}}{\rho ^b} \, C^{O_2b}_{total} \\ &= \frac{\rho ^{O_2}}{\rho ^b} \,( \, \underbrace{\alpha ^{O_2} p^{O_2b}}_{C^{O_2b}_{dis}} \, + \, \underbrace{N \, C^{Hb} \, S(p^{O_2b})}_{C^{O_2b}_{HbO_2}} \, ). \end{aligned} \end{aligned}$$

Here, $\rho ^{O_2}$ and $\rho ^b$ denote the density of oxygen and blood, respectively. Further, $C^{O_2b}_{total}$ is the total concentration of oxygen in the blood, which is the sum of the concentration of physically dissolved oxygen $C^{O_2b}_{dis}$ and the concentration of oxygen bound to hemoglobin $C^{O_2b}_{HbO_2}$. We use Henry’s law to calculate the concentration of physically dissolved oxygen using the solubility and partial pressure of oxygen in blood, $\alpha ^{O_2}$, $p^{O_2b}$, respectively. The concentration of oxygen bound to hemoglobin is calculated by the product of the Hüfner factor *N*, defined as the amount of oxygen per unit of hemoglobin, the saturation of hemoglobin with oxygen $S(p^{O_2b})$ and the concentration of hemoglobin $C^{Hb}$. The latter is calculated by

24 $$\begin{aligned} C^{Hb} = H \; MCHC, \end{aligned}$$

with *H* denoting the hematocrit value, defined as the volume fraction of red blood cells (RBCs) relative to the total blood volume, and *MCHC* representing the Mean Corpuscular Hemoglobin Concentration, which is a physiological parameter that quantifies the concentration of hemoglobin within individual RBCs. Further, the oxygen saturation of hemoglobin $S(p^{O_2b})$ is described by the Hill equation:

25 $$\begin{aligned} S\left( p^{O_2b}\right) = \frac{ \left( p^{O_2b}\right) ^{\gamma }}{\left( p^{O_2b}\right) ^{\gamma } + \left( p^{O_2b}_{50} \right) ^{\gamma }} \,, \end{aligned}$$

with the oxygen partial pressure at hemoglobin half-saturated $p^{O_2b}_{50}$ and the Hill exponent $\gamma$.

Coming to carbon dioxide, the missing link between the partial pressure and the concentration is given by the $CO_2$-dissociation curve. The relationship is quantitatively described by Loeppky et al. (1983) based on empirical data, given as

26 $$\begin{aligned} \begin{aligned}&\omega ^{CO_2b}(p^{CO_2b}) = \frac{\rho ^{CO_2}}{\rho ^b} \, C^{CO_2b} \\&= \frac{\rho ^{CO_2}}{\rho ^b} \, \left( 0.0301 p^{CO_2b} (1 + 10^{pH - 6.10} ) \right) \cdot 2.226 \\&\cdot \left( 1.0 - \frac{0.02924 \cdot Hb}{(2.244 - 0.422 \cdot S_{O_2} ) (8.740 - pH)} \right) , \end{aligned} \end{aligned}$$

where $C^{CO_2b}$ is the total concentration of carbon dioxide in the blood, *Hb* is the concentration of hemoglobin, *pH* the pH value and $S_{O_2}$ is the oxygen saturation of hemoglobin in the blood. The latter allows us to model the Halland-effect, the fact that deoxygenated blood can carry more carbon dioxide than oxygenated blood (West and Luks 2016). A similar equation was used by Busana et al. (2021).

### Larger airways and blood vessels as discrete reduced-dimensional networks

For gas exchange, the gases have to be transported to the exchange interface, i.e., the air-blood interface, modeled by or included in, respectively, the porous domain as described in the previous section. This is done on one side by the airways, which consist of branching tubes starting at the trachea and penetrating into the lungs, and on the other side by the blood vessels, which also form a series of branching tubes from the pulmonary artery to the capillaries and back to the pulmonary veins (West and Luks 2016). To maintain the hierarchical architecture of the larger airways and blood vessels, and thus the flow distribution within the lungs, their network structure is resolved in this study. However, we are not interested in the detailed flow field within individual airways and blood vessels but rather in the dynamics of the overall resulting flow. Therefore, a simplified, computationally efficient pipe-flow model is employed, dimensionally reducing the larger airways and blood vessels to their centerlines. The flow in the discrete airways and blood vessel segments is thereby described by the well-known Hagen-Poiseuille law, as it is often done for reduced-dimensional models of the vascular and respiratory systems, for example, in Berger et al. (2016), Kremheller et al. (2019) and Erbertseder et al. (2012).

Moreover, since most of the larger airways and blood vessels are surrounded by the tissue, they are affected by its movements. To account for this, we follow the approach by Kremheller et al. (2019) and embed the networks in the continous poroelastic domain. The discrete airways and blood vessels are embedded in the porous domain in a way that they follow the deformation of the underlying tissue. This means that every point of the discretely modeled airways and blood vessels corresponds to a point in the porous domain, and their movement is fully described by the movement of these coincident points. In the model presented here, we neglect the influences of the larger airways and blood vessels on the material properties of the tissue, i.e., stiffening effects, as well as changes from the tissue to the cross-sectional area of the larger airways and blood vessels, i.e., radial changes caused by the deformations of the tissue. The impact of these assumptions must be investigated in future work as the focus of this contribution lies in the development of the hybrid modeling approach, including the hierarchical structure of the larger airways and blood vessels and, thus, the flow distribution in the porous domain. Kremheller et al. (2019) presented the governing equations, that are used here for the flow and transport of respiratory gases in the discrete airways and blood vessels using the ALE formulation. We summarize them in the following sections.

#### Air and blood flow

The conservation of mass of a fluid phase $\hat{f}$ in the discrete airways and blood vessels $\Lambda _t$ written in ALE formulation is given by

27 $$\begin{aligned} \begin{aligned} A \frac{\partial \, v^t_t}{\partial t}&+ \frac{\partial \left( A \left( v^{\hat{f}} - v_t^t \right) \right) }{\partial t} \\ &- \frac{1}{\rho ^{\hat{f}}} \sum _{\kappa \in J_{n{\hat{f}}}} \overset{\kappa \rightarrow {\hat{f}}}{M} = 0 \, \, \, \, \, \, \, \, \text {on} \, \, \Lambda _t \times [t_0, t_e], \end{aligned} \end{aligned}$$

where we introduced the arc length coordinate *s* in the spatial configuration, or *S* in reference configuration to describe the location along the centerline, as it is usually used in such dimensionally-reduced models. Further, *A* describes the constant cross-sectional area, $v^{\hat{f}}$ the area averaged fluid velocity, $\rho ^{\hat{f}}$ the fluid density and $\overset{\kappa \rightarrow {\hat{f}}}{M}$ a generic mass transfer term, which is only used when coupling with the homogenized representatives in the porous medium and is therefore defined in Sect. 2.3. In addition, $v^t_t$ represents the tissue velocity vector projected in the tangential direction of the discretely modeled airway or blood vessel segment defined as

28 $$\begin{aligned} v^t_t = \boldsymbol{t_t} \cdot \frac{1}{a} \int \limits _{a} \boldsymbol{v}^t dA \approx \boldsymbol{t_t} \cdot \boldsymbol{v}^t, \end{aligned}$$

with the unit tangent vector $\boldsymbol{t_t}$. Further, we assume a stationary laminar flow through a cylindrical tube with constant radius *R* so the flow state in the reduced elements relative to the motion of the solid is described by the Hagen Poiseuille relationship

29 $$\begin{aligned} A \left( v^{\hat{f}} - v_t^t \right) = - \frac{\pi R^4}{8 \, \mu _{\hat{f}}} \left( \frac{\partial p^{\hat{f}}}{\partial t} \right) , \end{aligned}$$

with the dynamic viscosity of the fluid phase $\mu _{\hat{f}}$, which is assumed to be constant in the airways and blood vessels in the course of this study. This is a valid assumption for airflow; however, it neglects the non-Newtonian behavior of blood due to the presence of red blood cells. This effect, however, can be integrated into the model by using a more complex constitutive law for the viscosity, for example (Pries et al. 1992), if future research questions require it.

By inserting Eq. (29) into Eq. 27, we obtain the final equation governing the flow state in the discrete airways and blood vessels embedded in the surrounding tissue, which has to be solved for the primary variable $p^{\hat{f}}$:

30 $$\begin{aligned} \begin{aligned} \pi R^2 \frac{\partial \, v^t_t}{\partial t}&- \frac{\partial }{\partial t} \left( \frac{\pi R^4}{8 \, \mu _{\hat{f}}} \left( \frac{\partial p^{\hat{f}}}{\partial t} \right) \right) \\&- \frac{1}{\rho ^{\hat{f}}} \sum _{\kappa \in J_{n{\hat{f}}}} \overset{\kappa \rightarrow {\hat{f}}}{M} = 0 \, \, \, \, \, \, \, \, \text {on} \, \, \Lambda _t \times [t_0, t_e]. \end{aligned} \end{aligned}$$

#### Oxygen and carbon dioxide transport

The respiratory gases in the discrete airways and blood vessels are defined by their mass fractions $\omega ^{i\hat{f}}$ in the corresponding fluid phase $\hat{f} \ \epsilon \ \{ \hat{a},\hat{b}\}$, with $i \ \epsilon \ \{ O_2, CO_2 \}$. Their transport is governed by the instationary convection-diffusion–reaction equation given in ALE formulation as

31 $$\begin{aligned} \begin{aligned} \rho ^{\hat{f}} \pi R^2 \frac{\partial \omega ^{i\hat{f}}}{\partial t} \bigg |_{\boldsymbol{X}}&- \rho ^{\hat{f}} \frac{\pi R^4}{8 \, \mu _{\hat{f}}} \frac{\partial p^{\hat{f}}}{\partial t} \frac{\partial \omega ^{i\hat{f}}}{\partial t} \\&- \rho ^{\hat{f}} \frac{\partial }{\partial t} \left( \pi R^2 D^{i\hat{f}} \frac{\partial \omega ^{i\hat{f}}}{\partial t} \right) \\&+ \omega ^{i\hat{f}} \sum _{\kappa \in J_{n{\hat{f}}}} \overset{\kappa \rightarrow {\hat{f}}}{M} - \sum _{i \kappa \in J_{ni\hat{f}}} \overset{i \kappa \rightarrow i\hat{f}}{M} \\ &= 0 \, \, \, \, \, \, \, \, \text {on} \, \, \Lambda _t \times [t_0, t_e], \end{aligned} \end{aligned}$$

with the radius of the discretely modeled airway or blood vessel branch *R*, the dynamic viscosity $\mu _{\hat{f}}$, the pressure $p^{\hat{f}}$ and the density $\rho ^{\hat{f}}$ of the fluid phase, the diffusion coefficient of the respiratory gases $D^{i\hat{f}}$ and the mass transfer terms $\overset{\kappa \rightarrow {\hat{f}}}{M}$ and $\overset{i \kappa \rightarrow i\hat{f}}{M}$, which are only used when coupling with the homogenized representatives in the porous medium and are therefore defined in Sect. 2.3. Compared to the transport of the respiratory gases in the porous domain (see Eq. 18), there is only one change in the convection velocity, which is now given by the Hagen–Poiseuille law instead of the Darcy term.

### Coupling between homogenized and discrete representations of airways and blood vessels

The hybrid modeling approach in this study requires a coupling between the homogenized representations of the smaller respiratory and vascular units modeled through the porous domain (see Sect. 2.1) and the discrete representations of the larger airways and blood vessels modeled with a pipe-flow model (see Sect. 2.2). One type of coupling was already introduced in Sect. 2.2.1 by embedding the larger airways and blood vessels in the porous domain such that their deformation is fully described by the deformation of the underlying tissue.

**Fig. 4:**
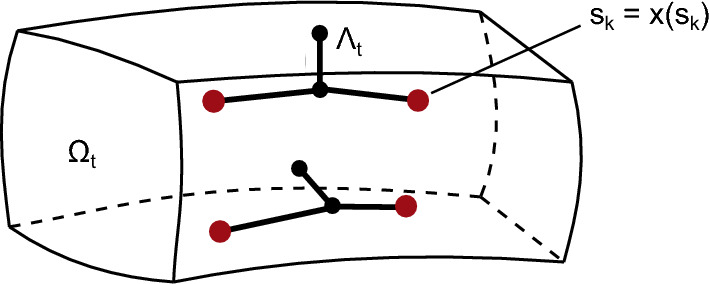
Sketch of exemplary discretely modeled pulmonary arteries and veins ($\Lambda _t$) embedded in the 3D porous domain ($\Omega _t$) with the coupling points, i.e., the most distal (away from the heart) node of the discrete embeddings, marked with red circles. Here, $s_k$ describes the spatial position of the coupling point in the discretely modeled blood vessels, and $x(s_k)$ is the spatial position of the coincident point in the 3D porous domain

In this section, we further couple air and blood flow as well as the transport of the respiratory gases in these phases between both domains. The basic idea thereby is mapping the fact that there is a continuity of the pressure, respectively the mass fraction of the respiratory gases, at the junction of the two domains, i.e., at the tips of the discretely modeled airway and blood vessel networks. These points are defined as the coupling points and marked in red in Fig. 4.

To ensure this continuity, we define the following constraint at the coupling points for the fluid flow (Eq. 32a) and the transport of the respiratory gases (Eq. 32b):

32a $$\begin{aligned} g_{\hat{f}-f} = p^{\hat{f}}(s_k,t) - p^{f}(x(s_k),t) = 0, \, \, \text {with} \; k \in J_{c}, \end{aligned}$$

32b $$\begin{aligned} g_{i\hat{f}-if} = \omega ^{i\hat{f}}(s_k,t) - \omega ^{if}(x(s_k),t) = 0, \, \, \text {with} \; k \in J_{c}. \end{aligned}$$

The spatial position of the coupling points is given by $x(s_k)$ in the porous domain, or $s_k$ in the discretely modeled networks (see Fig. 4). All coupling pair coordinates are collected in the coupling set $J_{c}$, which is a subelement of our set of neighboring phases and species in these phases introduced before. Equations 32a and 32b thus define "gaps" between the pressures and mass fractions in the homogenized and discretely modeled airways and blood vessels. The gaps have to vanish to ensure continuity. We enforce this constraint using a penalty method, which will be explained in more detail in Sect. 2.4.1. In contrast to the method for coupling the deformations of the discretely modeled airways and blood vessels and the surrounding tissue already introduced in Sect. 2.2.1, a bidirectional coupling is introduced here, meaning that the behavior in the discrete model influences the behavior in the porous domain and vice versa.

#### Remark

It should be noted here that to describe the fluid flow in the reduced airways and blood vessels as well as in the porous domain, we use a pure pressure-based formulation, meaning that only the pressure is solved as an independent primary variable. For this reason, only pressure continuity at the junction of the two domains is enforced for the coupling. The flow is not explicitly taken into account by an additional condition but is automatically determined by the formulated equations.

### Implementation

The derived modeling approach in this study describes a five-field initial boundary value problem composed of:

- solid (tissue) deformations (Eq. 8),
- fluid flow (air and blood) in the porous domain (Eq. 15),
- transport and exchange of the respiratory gases ($O_2$ and $CO_2$) in the porous domain (Eq. 18),
- fluid flow in the discretely modeled airways and blood vessels (Eq. 30) and
- transport of the respiratory gases ($O_2$ and $CO_2$) in these discretely modeled airways and blood vessels (Eq. 31),

in combination with coupling constraints for the fluid flow (Eq. 32a) and the transport of the respiratory gases (Eq. 32b) between the discrete and homogenized representations of airways and blood vessels.

This system of governing equations is solved for the unknown primary variables: the displacements $\boldsymbol{d}$ corresponding to Eq. 8, the pressures $p^f$ corresponding to Eq. 15, the mass fractions of the respiratory gases $\omega ^{i{f}}$ corresponding to Eq. 18 and the pressures $p^{\hat{f}}$ and mass fractions $\omega ^{i\hat{f}}$ corresponding to Eqs. 30 and  31, respectively. The equations and solver have been implemented in our open-source parallel multiphysics research code (4C 2025). We employ the finite element method for spatial discretization and the implicit one-step-theta-scheme for time discretization. To solve the coupled system of nonlinear PDEs, we use a fully monolithic solution algorithm (Kremheller et al. 2018) with a single Newton–Raphson loop in each time step. The resulting linear system of equations is composed of a $5 \, x \, 5$ block structure corresponding to our coupled five-field problem. It is finally solved using a standard generalized minimal residual method (GMRES) iterative solver with a preconditioner based on an algebraic multigrid (AMG) method and a block Gauss-Seidel (BGS) method (Verdugo and Wall 2016).

Special treatment is required in solving the instationary convection-diffusion–reaction equations for the transport and exchange of the respiratory gases, i.e., Eqs. 18 and 31. Under physiological conditions, the convective terms dominate the equations with Péclet numbers $\gg 1$, causing numerical instabilities when using the standard Galerkin method. To stabilize these equations, we employ the Streamline-Upwind Petrov-Galerkin (SUPG) method using a stabilization parameter as proposed by Taylor et al. (1998).

#### Treatment of 0D-3D coupling

For coupling the fluid flow and the transport of the respiratory gases between the discrete and homogenized representations of airways and blood vessels, we defined the constraint in Sect. 2.3 to ensure continuity of the pressures and the mass fractions at the junction of the two domains, see Eqs. (32a) and (32b). To enforce this constraint, we employ the penalty method—an approach classically used for contact or mesh-tying problems in the context of solid mechanics—with the penalty potential defined as

33 $$\begin{aligned} \Pi _{PM} = \frac{1}{2} \epsilon \int \limits _{\Gamma _c} \, g^2 dA \, \overset{\text {NTP}}{\approx } \, \frac{1}{2} \epsilon \sum _{k \in J_c} \, g_k^2 \, \, \, \, \, \, \, \, \text {on} \, \, J_c \times [t_0, t_e], \end{aligned}$$

where the term $\Gamma _C$ generally denotes the coupling area, which in our model reduces to a sum over the discrete coupling points, i.e., the spatial positions of all end-point nodes of the discretely modeled airway and blood vessel networks and their coincident coordinates in the porous domain. They are combined in the active coupling set $J_c$. In contrast to classical contact problems in solid mechanics where the actual active contact surface is generally unknown a priori and will likely change over time, in our case, the problem is much simpler as our coupling points are known in advance and fixed. This means that a search algorithm only has to find the coupling points once in a preprocessing step and that these points do not vary in time as a result of our coupling constraint that the larger airways and blood vessels follow the deformation of the surrounding tissue. The fact that there is no relative movement between the discrete and homogenized domain (see Sect. 2.2) greatly reduces the computational costs. The constraint at the coupling points are, therefore, treated as Dirichlet or Neumann boundary conditions and prescribed in advance. Further, *g* generally denotes the constraint, representing Eqs. (32a) and (32b) and $\epsilon$ define the penalty parameter, which controls how the constraint is fulfilled. Because all constraints are always evaluated between a discrete blood vessel or airway node and the corresponding point in the 3D porous domain that has the same spatial location, this coupling discretization is referred to as a node-to-point (NTP) formulation.

Variation of Eq. (33) leads to the contribution to the weak form:

34 $$\begin{aligned} \delta \Pi _{PM} = \epsilon \sum _{k \in J_c} \, g_k \, \delta g_k, \end{aligned}$$

with the definition of the constraint for coupling the fluid flow (Eq. 32a) resulting in:

35 $$\begin{aligned} \begin{aligned}&\delta \Pi _{PM, \, \hat{f}-f} \\ &= \epsilon \sum _{k \in J_c} \left( p^{\hat{f}}(s_k) - p^{f}(x(s_k))\right) \delta \left( p^{\hat{f}}(s_k) - p^{f}(x(s_k))\right) \\&= \epsilon \underbrace{\sum _{k \in J_c} \delta p^{\hat{f}}(s_k) \, \, \left( p^{\hat{f}}(s_k) - p^{f}(x(s_k))\right) }_{Contribution \; to \, Eq. 30} \\&\, \, \underbrace{ - \;\epsilon \sum _{k \in J_c} \delta p^{f}(x(s_k)) \, \, \left( p^{\hat{f}}(s_k) - p^{f}(x(s_k))\right) }_{Contribution \; to \, Eq. 15}, \end{aligned} \end{aligned}$$

and the definition of the constraint for coupling the transport of the respiratory gases (Eq. 32b) in:

36 $$\begin{aligned} \begin{aligned}&\delta \Pi _{PM, i\hat{f}-if} \\ &= \epsilon \underbrace{\sum _{k \in J_c} \delta \omega ^{i\hat{f}}(s_k) \, \, \left( \omega ^{i\hat{f}}(s_k) - \omega ^{if}(x(s_k))\right) }_{Contribution \; to \; Eq. 31} \\ &\, \, \underbrace{ - \;\epsilon \sum _{k \in J_c} \delta \omega ^{if}(x(s_k)) \, \, \left( \omega ^{i\hat{f}}(s_k) - \omega ^{if}(x(s_k))\right) }_{Contribution \; to \, Eq. 18}. \end{aligned} \end{aligned}$$

The test functions $\delta p^{\hat{f}}, \delta \omega ^{i\hat{f}} \in H_0^1(\Lambda _t)$ and $\delta p^{f}, \delta \omega ^{if} \in H_0^1(\Omega _t)$ of the discrete and homogenized domain, respectively, are used. Equations 35 and 36 show the contributions to the weak formulations of the corresponding equations. These discrete coupling contributions exactly balance each other, ensuring global conservation of mass. It has to be noted that due to our non-conforming coupling, the coupling point does not have to coincide with a node in the mesh of the porous domain but can be located anywhere in the element. We use a local Newton iteration for mapping the spatial coupling coordinate $x(s_k)$ to the corresponding parameter coordinate employing the isoparametric concept.

A major advantage of the method is the straightforward implementation. It is, however, accompanied by the challenge of selecting a suitable penalty parameter that fulfills the constraint sufficiently accurately without overconstraining the problem. In this context, we should keep in mind that we couple a resolved and a homogenized model domain. As a result, there is no physically "correct" solution, unlike, for example, the more intuitive no-penetration constraints in solid mechanics. This makes the requirement for an exact fulfillment of the constraint less stringent, in our opinion. Nevertheless, to overcome this issue, alternative constraint enforcement strategies, such as the Lagrange multiplier method, could be considered in future work. However, this lies beyond the scope of the present study.

##### Remark

The coupling contributions in Eqs. 35 and 36 may be interpreted as mass exchange terms between the discrete and homogenized representations with the penalty parameter serving as a permeability at the interface, similar to the approach of Vidotto et al. (2019). Indeed, for the fluid flow, the coupling contribution at the tips of the discretely modeled larger airways and blood vessels has the form of a nonlinear Neumann boundary condition, making it explicit that by only enforcing pressure continuity between the domains in our formulation, it implicitly also couples the flow.

##### Remark

For reasons of clarity, the time dependence of the variables has been omitted in this section. All coupling contributions, both to the equations for fluid flow and the transport of the respiratory gases in these fluid phases in the porous domain and the discretely modeled networks (see Eqs. 35 and 36), are evaluated at the intermediate time instant $t_{n+\theta }$. Additionally, the contribution to the scalar transport equations is scaled by the time factor $-\Delta \, t$, resulting from the instationarity, i.e., the time derivative of the mass fraction of the gases.

## Numerical examples

In this study, we show two numerical examples. First, we present a simple quasi two-dimensional example to demonstrate the capability of our derived modeling approach, as well as the correctness of the numerical methods and their implementation in a well-defined environment. Second, we present a three-dimensional example using a patient-specific geometry of the upper left lung lobe to prove the general applicability of our approach to realistic clinical scenarios.

### Idealized rectangular lung (quasi 2D) with heterogeneous tissue stiffness

**Fig. 5:**
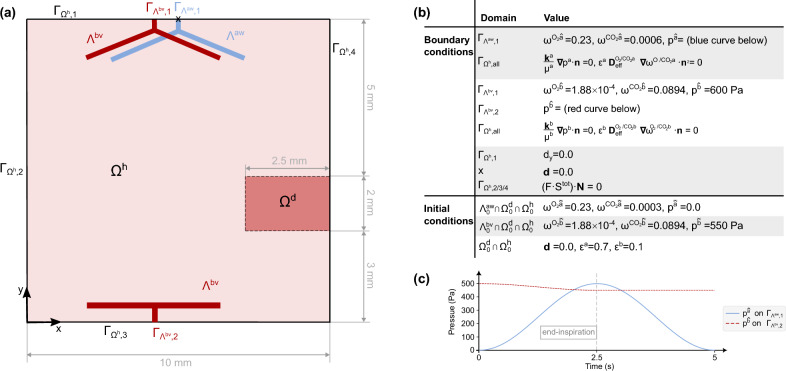
Simulation setup. The geometry (not to scale) is shown on the left (**a**) and the initial and boundary conditions on the right (**b**), with the time-dependent boundary conditions at the airway inlet and at the venous end shown below (**c**)

In this section, we present a quasi two-dimensional academic example that studies the behavior of two terminal acinar units in the lungs during mechanical ventilation. The aim of this example is to verify the correctness of the presented numerical methods and their implementation within a simple well-defined setting, as well as to demonstrate the capability of our proposed framework to model the coupling between discrete and homogenized representations of airways and blood vessels, the impact of ventilation on the pulmonary circulation and gas exchange dynamics. The simulation setup with the initial and boundary conditions is sketched in Fig. 5.

We analyze a rectangular porous domain $\Omega$ of 10 mm $\times \, 10$ mm intending to represent smaller respiratory and vascular units of two acinar units in the lungs. We consider a healthy and a diseased area, $\Omega ^h$ and $\Omega ^d$, respectively, which differ in terms of increased tissue stiffness (Young’s modulus $E^h = 3000$ Pa and $E^d = 4000$ Pa). This subdivision allows us to investigate how lung deformation influences the volume fraction of smaller vascular units (see Sect. 2.1.3). Our porous domain is supplied by two discretely modeled major airways $\Lambda ^{aw}$ (shown in blue), arteries and veins $\Lambda ^{bv}$ (shown in red), which are coupled with the porous domains at the network end-points through our proposed approach described in Sect. 2.3.

In this example, we mimic that our domain is mechanically ventilated. We, therefore, prescribe a pressure-controlled boundary condition at the airway inlet of $p^{\hat{a}} = 250 \cdot ( 1- \text {cos}( \pi \cdot t \cdot 0.4))$ Pa. In total, we simulate one breathing cycle with a duration of 5 seconds. To model the transport and exchange of the respiratory gases, at the airway inlet, we additionally prescribe the mass fraction of oxygen $\omega ^{O_2\hat{a}} = 0.23$ corresponding to 122  mmHg and carbon dioxide $\omega ^{CO_2\hat{a}} = 0.0003$ corresponding to 22.5  mmHg, as they are in the atmosphere. Changes in the partial pressure during inspiration due to changes in temperature and humidity are not considered.

For the pulmonary circulation, we simulate the physiological condition in which we have continuous blood flow into the porous domain through the larger arteries and out (back to the heart) through the larger veins. Therefore, we set a Dirichlet boundary condition for the pressure at the artery inlet of $p^{\hat{b}} = 600$ Pa and a lower pressure boundary condition at the vein outlet as plotted in Fig. 5c. The latter is designed to let the pressure of blood drop below the pressure of air in the porous domain near the venous network during end-inspiration. This allows us to study the pressure dependence of our newly introduced evolution equation (see Sect. 2.1.3). In the porous domain, we assume an initial volume fraction of blood $\varepsilon ^b_0=0.1$ (Itoh et al. 2004) and the parameters describing its evolution $k_J$ and $k_p$ to $-1/3$ and $-2/3$, respectively. Further, we set the mass fraction of oxygen $\omega ^{O_2\hat{b}} = 1.88 \times 10^{-4}$ and carbon dioxide $\omega ^{CO_2\hat{b}} = 0.0894$ at the artery inlet as they are in the main pulmonary artery as Dirichlet conditions. At all boundaries of the porous domain, we prescribe no normal flux of fluid or respiratory gases to map the lung pleural boundary.

Concerning the displacement, we fix the rectangle at the airway inlet in all spatial directions and only allow tangential movement on the upper boundary ($d_y = 0.0$ on $\Gamma _{\Omega ^h,1}$). At all other boundaries, we prescribe no normal force to approximate zero pleural pressure. The initial porosity is set to $\varepsilon _0 = 0.8$ in the entire domain, which is within the range presented in Peyraut and Genet (2024).

**Fig. 6:**
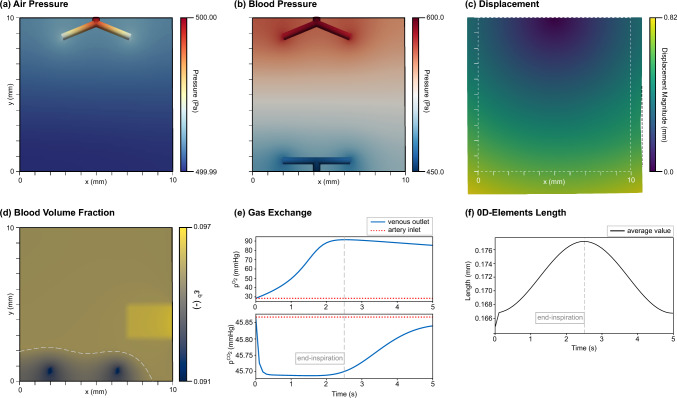
Simulation results: (**a**)–(**d**) show the results at end-inspiration, after 2.5 s: (**a**) depicts the pressure in the respiratory part in reference configuration, (**b**) the pressure in the circulatory part in reference configuration, (**c**) the displacements (scaled by a factor of two for better visibility, with the dashed white line indicating the initial configuration), and (**d**) the volume fraction of blood in reference configuration (where the dashed line indicates that below this line the pressure of air exceeded the pressure of blood); (**e**) compares the partial pressure of oxygen (upper plot) and carbon dioxide (lower plot) between the artery inlet and the venous end and (**f**) shows the average length of all reduced dimensional elements during the entire breathing cycle

Both domains are discretized independently. We performed a mesh refinement study and identified a regular mesh using $50 \times 50$ bilinear elements for the porous domain to yield a good trade-off between computational efficiency and accuracy. The major airway, artery and vein networks are each discretized using 30 linear elements with a constant diameter of $D = 500 \, \mu m$, referring to a physiological size of terminal airways (Haefeli-Bleuer and Weibel 1988) and blood vessels (Formaggia et al. 2009). This rather fine discretization of the linear elements is used to reduce the element Péclet number. For time discretization, we employ the implicit Backwards Euler scheme and a time step size of $\Delta \, t = 0.1$ s. We use a penalty parameter of $\epsilon _a = 1.0 \times 10^{-4}$ ${\textrm{m}^2 \, \textrm{Pa}^{-1} \, \textrm{s}^{-1}}$ for coupling air pressure, $\epsilon _b = 1.0 \times 10^{-6}$ ${\textrm{m}^2 \, \textrm{Pa}^{-1}\, \textrm{s}^{-1}}$ for coupling the blood pressure and $\epsilon _{O_2, CO_2} = 1.0 \times 10^{-5}$ ${\textrm{m}^2 \, \textrm{s}^{-1}}$ for coupling the respiratory gases, which are within the range proposed by Kremheller et al. (2019). The material parameters used in this analysis are listed in the Appendix A.

Figure 6a illustrates the pressure distribution within the respiratory system at end-inspiration. The porous domain exhibits an approximately uniform pressure of 500 Pa, corresponding to the prescribed pressure at the airway inlet. It shows that the pressure from the discretely modeled larger airways successfully propagates to the distal respiratory units within the acinus. A similar behavior is observed in the pulmonary circulation. A pressure gradient has developed from the major discretely modeled arteries to the major discretely modeled veins, depicted in Fig. 6b. It drives physiological blood flow through the smaller vascular units in the porous domain. In both systems, the pressure values at the network end-points are identical in the discrete model and the corresponding location in the porous domain, verifying our coupling approach. The used penalty parameters show good fulfillment of the constraints. Although the choice of the penalty parameter is somewhat arbitrary, we tested different values (ranging from $1.0 \times 10^{-8}$ to $1.0 \times 10^{-3}$ $\textrm{m}^2 \, \textrm{Pa}^{-1} \, \textrm{s}^{-1}$), which had no significant impact on the analyzed pressure field and are therefore not shown here.

The pressures in the smaller respiratory and vascular units act on the solid tissue and lead to an expansion of the domain, as depicted in Fig. 6c. The initial (undeformed) configuration is marked by the white dashed line. Tissue deformation is primarily driven by the nearly uniform air pressure field, while the contribution of the blood pressure is quite small in this example due to its low volume fraction. In this case, it might be justified to neglect the contribution from the blood pressure in calculating the tissue pressure in Eq. (5), however, its contribution should be analyzed in patient-specific simulations in future studies. The deformation of the porous domain affects the deformation of the embedded, discretely modeled, larger airways and blood vessels. As depicted in Fig. 6f, their length increases during inspiration, when the tissue expands, and decreases during expiration when the tissue contracts. The time course directly reflects the applied pressure boundary condition at the airway inlet, which drives the respiratory cycle. The sharp initial rise results from the imposed initial conditions of zero deformation paired with an already elevated blood pressure. The overall change in length between end-expiration and end-inspiration is about 6 $\%$, which has a negligible impact on the results for this simple example, however, its influence should be investigated in larger lung regions with larger airway and blood vessel trees in future studies. Further, the locally reduced tissue elastance in the diseased region $\Omega ^d$ (lower right) leads to a slightly reduced expansion, as visible in Fig. 6c. This effect becomes more evident in Fig. 6d, which shows the volume fraction of blood in the porous domain. Two effects of our newly introduced evolution equation of blood are highlighted: first, the blood volume fraction is deformation-dependent and shows higher values in the less deformed region. Lung expansion during inspiration compresses the smaller blood vessels in the acinar units, which reduces their blood content. Second, a marked drop in blood volume fraction is observed near the discretely modeled veins, below the dashed white line—-representing the region where air pressure exceeds blood pressure. The higher air pressure in the alveoli squeezes the smaller surrounding blood vessels and causes them to collapse. This effect has to be considered during recruitment maneuvers in the clinic, where high air pressures are typically applied aiming to open collapsed units. In this study, the parameters governing the evolution equation of the volume fraction of blood have been chosen heuristically. Their effects and identification clearly need further analysis, however it is not the focus of this study.

Further, this example models the full gas exchange process in the lungs. Air and blood flow in the larger airways and blood vessels transport the respiratory gases to the exchange interface in the acinar domain. Figure 6e compares the partial pressures of oxygen and carbon dioxide at the artery inlet and (after flowing through the porous domain) at the vein outlet during the entire breathing cycle. The pulmonary circulation is initialized with fully degassed blood (low oxygen and high carbon dioxide concentrations). During the simulation, we could qualitatively capture the general gas exchange process: oxygen is taken up, and carbon dioxide is released as blood traverses the porous domain. Again, we emphasize that the simulation results are not designed to be compared to physiological values quantitatively. In particular, the partial pressures of oxygen and carbon dioxide equilibrate between air and blood at levels that differ from typical physiological values, mainly due to the simplified geometry design and idealized initial and boundary conditions.

### Three-dimensional example using a patient-specific geometry

It is well-known that lung diseases exhibit heterogeneous patterns that vary among patients. Thus, treatment strategies are often not universally applicable but should be tailored to the individual patient. We, therefore, plan to apply our proposed modeling approach to patient-specific applications in future studies. As a first step, we present an example with a patient-specific geometry of the left top lung lobe in this section. This example aims to demonstrate the principal applicability of our approach to realistic three-dimensional problems; it is not, however, intended to provide physiologically interpretable results at this stage.

**Fig. 7:**
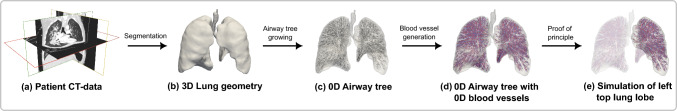
Workflow of patient-specific geometry generation. We use (**a**) patient CT data to (**b**) segment the lung lobes and visible first airway generations. Based on this, we use (**c**) algorithms to generate the peripheral larger airway branches (Ismail et al. 2013; Roth et al. 2017; Tawhai et al. 2000) and from them, (**d**) the larger pulmonary blood vessels (Ismail et al. 2013; Ismail 2014). We perform a simulation using (**e**) the geometry of the left top lung lobe

The workflow to generate the patient-specific geometry is based on our previous publications (see, for example, Ismail et al. (2013), Geitner et al. (2024) and Roth et al. (2017)). It is sketched in Fig. 7 and will be briefly explained in the following.

**Fig. 8:**
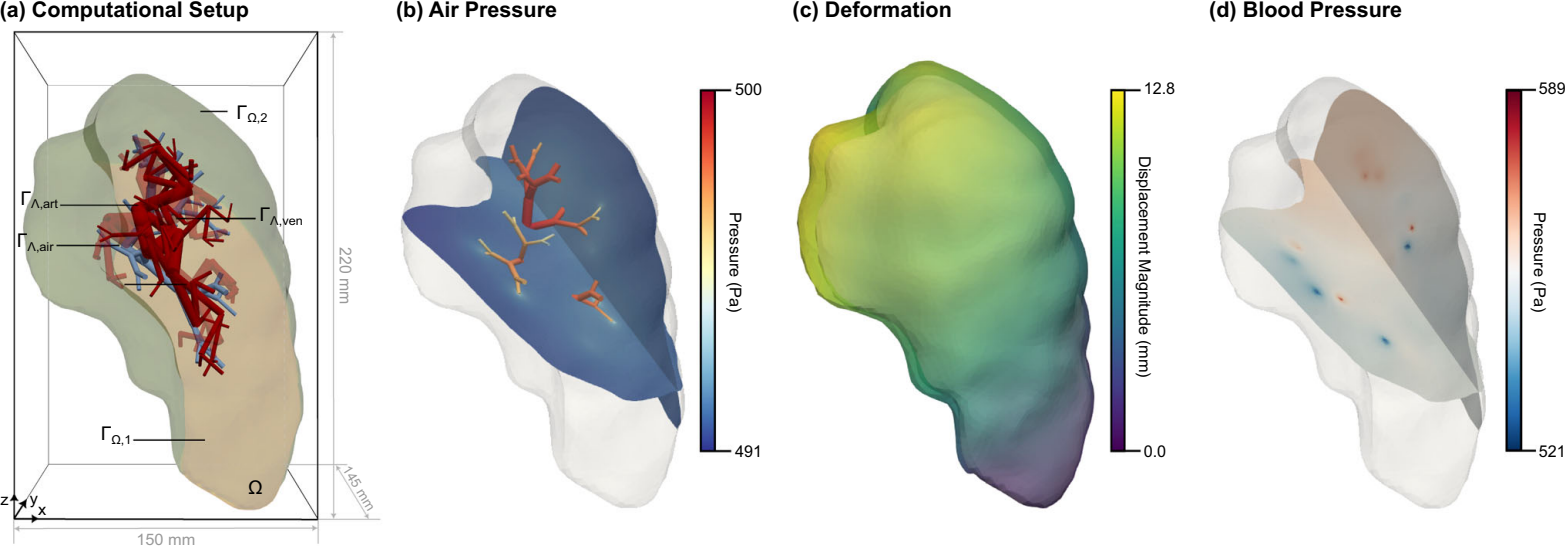
Simulation setup (**a**) showing the left top lung lobe including five generations of discretely modeled larger airways (shown in blue), arteries (shown in red), and veins (shown in light red). Simulation results (**b**)–(**d**) at end-inspiration ($t = 2.5$ s): (**b**) air pressure in reference configuration; (**c**) deformation state (including initial configuration shown in gray); (**d**) blood pressure in reference configuration

The only clinical input required is a single three-dimensional CT scan of the patient’s thorax (Fig. 7a). Here, we use a CT scan acquired during a clinical study (Becher et al. 2021) at a predefined positive end-expiratory pressure (PEEP), corresponding to the end-expiratory lung volume. This scan is utilized to segment the five lung lobes using the software package Mimics (Materialise, Leuven, Belgium), providing the geometry of our porous domain (Fig. 7b). Additionally, the centerlines of the visible first airway generations—typically reaching from the trachea to the 5th generation (where the airways enter the lobes)—are extracted from the medical image. Due to the resolution limitations of CT scans, peripheral airway branches within the individual lobes are not directly visible and are thus generated using a space-filling algorithm based on the work of Ismail et al. (2013), Roth et al. (2017) and Tawhai et al. (2000) (shown in Fig. 7c). To generate the major pulmonary blood vessels, specifically the arterial and venous networks, we employ an anatomically informed algorithm presented in (Ismail 2014, pp. 23–25) which is based on the airway tree geometry. In this study, we slightly modified the algorithm to ensure that all vessels live inside the lobe. The generated network structure is illustrated in Fig. 7d. In this example, we restrict our analysis to the deformation and flow dynamics within the left top lung lobe as a "proof of principle" (see Fig. 7e). It includes five generations of discretely modeled larger airways (shown in blue), arteries (shown in red), and veins (shown in light red) starting inside the lung lobe. The simulation setup is depicted in Figure 8a.

The boundary and initial conditions are again set to mimic that our lung lobe is ventilated. At the airway inlet $\Gamma _{\Lambda ,\text {air}}$, we prescribe again a Dirichlet boundary condition of the pressure ($p^{\hat{a}} = 250 \cdot (1 - \text {cos}(\pi \cdot t \cdot 0.4))$ Pa) simulating one breathing cycle with a duration of 5 seconds. For pulmonary circulation, a constant Dirichlet boundary condition for the pressure is set at the artery inlet $\Gamma _{\Lambda ,\text {art}}$ ($p^{\hat{b}} = 600$ Pa) and the vein outlet $\Gamma _{\Lambda ,\text {ven}}$ ($p^{\hat{b}} = 500$ Pa). This setup mimics physiological blood flow, directing flow from the larger arteries through the smaller vessels in the porous domain (assuming an initial volume fraction $\varepsilon ^b_0 = 0.1$ and constant absolute blood volume with $k_J = - 1.0$) and out through the larger vein network. At the outer surface of the lung lobe ($\Gamma _{\Omega ,1} \cup \Gamma _{\Omega ,2}$), a Neumann boundary condition is imposed with zero normal force and fluid flux to approximate the lung pleural boundary assuming a homogeneous zero pleural pressure. Finally, to spatially define our domain, we fix the lobe-lobe boundary $\Gamma _{\Omega ,1}$ (orange surface in Fig. 8a, boundary to the left bottom lobe of the lungs) in all spatial directions.

Again, we performed a mesh refinement study and use a non-conforming mesh to discretize both domains, which provides a major advantage in handling the intricate network structure of the larger airways and blood vessels. Specifically, the porous domain is discretized using 267, 577 quadratic tetrahedral elements, while the discretely modeled networks, each comprising 62 branches, are discretized with 62 linear elements, respectively. For the time discretization, we employ the implicit Backwards Euler scheme with a time step $\Delta \, t = 0.1$ s. The used parameters are shown in the Appendix A. To hold this example very simply, we have not yet considered the respiratory gases and have neglected the influence of blood pressure on the deformation. The simulation results at end-inspiration ($t = 2.5$ s) are shown in Fig. 8b–d, with the pressure distribution in the respiratory system depicted in Fig. 8b. For better illustration, we show two slices of the lung lobe, whereby the gray cover indicates the outer surface. The prescribed pressure increase at the airway inlet again propagates to the peripheral units inside the lobe. The local inflows (pressure increases) into the porous medium at the network end-points resulting from the coupling can be clearly seen. The pressure in the respiratory system leads to an inflation of the entire domain with a resulting deformation shown in Fig. 8c. The initial configuration is shown in light gray. As the lung lobe expands, the embedded discretely modeled airways and blood vessels are also moved along with it. Their length changes only slightly—on average by about $8 \%$ between end-expiration and end-inspiration, which has little effect on the overall results and is therefore not shown here. However, since it strongly depends on the selected material parameters and boundary conditions, its impact should be investigated within a patient-specific setup in future studies.

Further, Fig. 8d shows the pressure distribution of blood within the lobe (again, slices of the lung lobe whereby the gray cover indicates the outer surface). For better illustration, we do not show the discretely modeled larger artery and vein network here. In the porous domain, we again see local pressure increases and decreases resulting from the coupling with the larger arteries and veins, respectively. Again a gradient from the arterial inlet to the venous outlet has developed, reflecting the physiological blood flow from larger arteries through the porous domain and out via the discrete venous network.

## Discussion

In clinical practice, defining ventilation strategies that keep lung tissue in a healthy state while providing sufficient gas exchange remain a major challenge. This difficulty stems from the complex, tightly coupled, yet still insufficiently understood processes within the lungs. A deeper understanding of the coupled interactions among ventilation, tissue mechanics, perfusion, and gas exchange is therefore critical to improving patient care.

To achieve this goal, in this study, we derived a comprehensive, physics-based computational model of the human lungs that bidirectionally couples the respiratory system with the pulmonary circulation including gas exchange. We developed a mixed-dimensional approach that models the major airways and blood vessels as discrete networks, which are embedded into a multiphase porous medium. The porous domain captures the coupling of airflow, blood flow, and tissue deformation at the alveolar level in a consistent continuum mechanical way. To close the system of equations, we proposed an evolution equation describing the kinematics of the volume fraction of blood during breathing based on two known phenomena: the smaller vascular units depend on the deformation of the surrounding tissue, as well as on the pressure within the surrounding alveoli. We tested the full modeling approach using a simple academic example, demonstrating that the proposed framework captures key features of lung physiology: the hierarchical flow distribution in the lungs, the coupled interaction of the respiratory and circulatory systems, and the qualitative process of gas exchange.

A major advantage of our model is the bidirectional coupling between the respiratory and circulatory systems. Unlike approaches that rely on precomputed ventilation patterns to estimate blood flow it allows us to study how changes in one system affect the other. We can, thus, use the model to simulate the effects of mechanical ventilation on cardiac function and vice versa in future studies. The introduction of blood as a second fluid phase in the porous domain required the formulation of an evolution equation for the blood volume fraction in order to close the system of governing equations. Although this closing relation is motivated by known physiological mechanisms, it introduces two additional parameters governing the deformation- and pressure-dependence of the blood volume fraction. The identification and calibration of these parameters may be challenging, particularly for patient-specific applications. The impact of these newly introduced parameters should therefore be investigated further, for example by means of uncertainty quantification, as proposed by Wirthl et al. (2023) and Nitzler et al. (2022). Moreover, the current evolution equation does not yet explicitly account for the effect of transmural pressure; instead, it focuses on the dominant mechanism arising when airway pressure exceeds blood pressure. Incorporating transmural pressure effects would require additional model parameters. The effect of this simplification should be examined in future studies.

Compared to existing approaches in the literature modeling lung ventilation and perfusion (Clark et al. 2011; Burrowes et al. 2011, 2017), which treat each respiratory unit as independent, a key advantage of our porous medium approach is its ability to capture the connectivity between neighboring respiratory units. This allows us to not only mimic the effect of localized disease conditions on global lung function but also to explore how this disease can impact the nearby healthy, well-ventilated regions—-a central point in the development of VILI as strain hotspots are often observed at margins between ventilated and collapsed areas (Mead et al. 1970).

Further, we want to stress that our modeling approach is able to direct airflow and blood flow in the respiratory zone modeling the lungs’ hierarchical structure. This is achieved by coupling the porous domain with discrete representations of the larger airways and blood vessels. It allows us to simulate realistic flow distributions in the lungs and to capture the effects of regional airway constrictions and blood vessel occlusions on the overall lung function in future studies. A major advantage of our coupling approach is the ability to use non-matching discretization for the porous domain and discrete networks, which makes it much easier to deal with the complex network structures of the lungs. However, the coupling of fluid flow and species transport at the network end-points is represented by a point-wise (NTP) approach in this study. This singularity may cause convergence issues in more complex applications. A possible remedy would be to distribute the coupling over a finite region in the porous domain, i.e., the outer surface area of the discretely modeled networks, similar to the lateral surface coupling approach presented by (Kremheller, p. 66). Additionally, the discretely modeled larger airways and blood vessels are embedded into the porous domain in a way that they follow the deformation of the surrounding tissue. However, radial expansion and contraction—which influence flow resistance—are not yet considered in this study. Their effect should be investigated in future work.

Further, we want to emphasize that our modeling approach captures the full gas exchange process of the respiratory gases oxygen and carbon dioxide. Compared to existing publications that only model oxygen transport and exchange (Burrowes et al. 2011; Ismail 2014; Roth et al. 2017; Burrowes et al. 2017), we also consider carbon dioxide, which removal is a crucial issue in the clinic.

To already give a short outlook on the usability of our approach, we showed an example with a patient-specific geometry of the left top lung lobe. We presented a workflow for generating patient-specific geometries from CT data, including the network structure of the larger airways and blood vessels. While this simplified example was not intended to produce quantitatively interpretable results for clinical validation, it illustrates the feasibility of our approach using patient-specific geometries. It proves the model’s capability to handle the coupling with the complex network structure of the larger airways and blood vessels. The current model formulation does not yet incorporate the effects of gravity or pleural pressure. Extending the framework to account for these factors constitutes an important direction for future work and will enable the investigation of patient-specific, heterogeneous pulmonary diseases.

Finally, we want to highlight that our modeling approach presents a flexible framework that can easily be extended to include exchange between phases, as well as additional phases so that pathological conditions such as transcapillary leakage or pulmonary edema—hallmarks of VILI—can be studied in future stages. We consider our model to be a promising base for investigating clinically relevant questions in a predictive and patient-specific manner, which will contribute to improved treatment in respiratory care.

## Conclusion

In this study, we derived a mixed-dimensional modeling approach of the human lungs that bidirectionally couples the respiratory system with the pulmonary circulation, including gas exchange. Motivated by the anatomical structure of the lungs, we model the major airways and blood vessels as discrete networks, which are embedded into a multiphase porous medium. The porous medium captures airflow, blood flow, and tissue deformation at the alveolar level in a consistent continuum mechanical framework. Additionally, our model incorporates the transport and exchange of the respiratory gases oxygen and carbon dioxide. The model allows studying key features in lung mechanics, including the hierarchical distribution of flow, the coupled interactions between ventilation, tissue deformation, and perfusion, and their impact on gas exchange—all in a predictive and patient-specific manner. Thus, the model can be used to analyze the clinically significant effects of ventilation on tissue strain and gas exchange dynamics. We consider our modeling approach an important step toward advancing the understanding of processes involved in mechanical ventilation and, ultimately, improving patient care in respiratory medicine.

## Data Availability

All data generated during this study are available from the corresponding author upon reasonable request.

## Appendix A: Simulation parameters

This section provides the parameters used in the simulations. The parameters are divided into four categories: lung tissue, airflow, blood flow, and gas exchange.

Tables 1, 2, 3, 4.

**Table 1:** Parameters of the lung tissue

Symbol	Parameter	Value	Units	References
$E^h$	Young’s modulus healthy	3000	Pa	Assumed^1^
$E^d$	Young’s modulus diseased	4000	Pa	Assumed
$\nu$	Poisson’s ratio	0.3	–	Yoshihara et al. (2017)
$\rho ^t$	Density	1000	$\textrm{kg}\,\textrm{m}^{-3}$	Yoshihara et al. (2017)

^1^ Within the range of values reported in Yoshihara et al. (2017) and Birzle et al. (2018)

**Table 2:** Parameters of airflow

Symbol	Parameter	Value	Units	Reference
$\rho ^a, \rho ^{\hat{a}}$	Density of air	1.225	$\textrm{kg}\,\textrm{m}^{-3}$	Known
$\mu ^a, \mu ^{\hat{a}}$	Dynamic viscosity of air	$1.5 \times 10^{-5}$	$\textrm{kg}\,\textrm{m}^{-1}\,\textrm{s}^{-1}$	Known
$k^a$	Permeability of air	$1.2 \times 10^{-9}$	$\textrm{m}^{2}$	Degroot and Straatman (2012)

**Table 3:** Parameters of blood flow

Symbol	Parameter	Value	Units	Reference
$\rho ^b, \rho ^{\hat{b}}$	Density of blood	1030	$\textrm{kg}\,\textrm{m}^{-3}$	Known
$\mu ^b, \mu ^{\hat{b}}$	Dynamic viscosity of blood	0.004	$\textrm{kg}\,\textrm{m}^{-1}\,\textrm{s}^{-1}$	Known
$k^b$	Permeability of blood	$1.5 \times 10^{-10}$	$\textrm{m}^{2}$	Assumed^1^

^1^ This value is estimated based on permeability values used for lung capillaries in Erbertseder et al. (2012). To additionally account for the presence of supplying larger blood vessels within the vascular units, it is scaled by four orders of magnitude, resulting in a value comparable to the permeability of air

**Table 4:** Parameters of gas exchange

Symbol	Parameter	Value	Units	Reference
$\rho ^{O_2}$	Density of oxygen	1.429	$\textrm{kg}\,\textrm{m}^{-3}$	Known
$\rho ^{CO_2}$	Density of carbon dioxide	1.98	$\textrm{kg}\,\textrm{m}^{-3}$	Known
$D^{CO_2a}, D^{CO_2\hat{a}}$	Diffusion coefficient of carbon dioxide in air	$1.48 \times 10^{-5}$	${\textrm{m}^{2} \;\textrm{s}^{-1}}$	Known
$D^{CO_2b}, D^{CO_2\hat{b}}$	Diffusion coefficient of carbon dioxide in blood	$1.48 \times 10^{-9}$	${\textrm{m}^{2} \;\textrm{s}^{-1}}$	Known^1^
$D^{O_2a} D^{O_2\hat{a}}$	Diffusion coefficient of oxygen in air	$1.76 \times 10^{-5}$	${\textrm{m}^{2} \;\textrm{s}^{-1}}$	Known
$D^{O_2b} D^{O_2\hat{b}}$	Diffusion coefficient of oxygen in blood	$2.18 \times 10^{-9}$	${\textrm{m}^{2} \;\textrm{s}^{-1}}$	Known
$\alpha ^{O_2}$	Solubility of oxygen in water	$0.21 \times 10^{-6}$	$\textrm{Pa}^{-1}$	Klinke et al. (2010)^2^
$\alpha ^{CO_2}$	Solubility of carbon dioxide in water	$5.06 \times 10^{-6}$	$\textrm{Pa}^{-1}$	Klinke et al. (2010)^2^
$N \cdot MCHC$	Max. hemoglobin-bound oxygen	0.5	−	Possenti et al. (2021)
*H*	Discharge hematocrit	0.5	−	Possenti et al. (2021)
$p_{50}^{O_2}$	Partial pressure of oxygen at 50 % oxygen Saturation	3600	Pa	Klinke et al. (2010)
$\gamma$	Hill exponent	3	Pa	Possenti et al. (2021)
*d*	Thickness of blood gas barrier	$1.0 \times 10^{-6}$	m	Klinke et al. (2010)
$D^{O_2t}$	Diffusion coefficient of oxygen in tissue	$2.41 \times 10^{-9}$	$\textrm{m}^{2}\,\textrm{s}^{-1}$	Possenti et al. (2021)
$D^{CO_2t}$	Diffusion coefficient of carbon dioxide in tissue	$2.41 \times 10^{-9}$	$\textrm{m}^{2}\,\textrm{s}^{-1}$	Assumed^3^
*S*	Gas exchange surface	120	$\textrm{m}^{2}$	Klinke et al. (2010)
*V*	Total lung capacity	$6.2 \times 10^{-3}$	$\textrm{m}^{3}$	Known^4^
$\epsilon ^{Healthy}$	Healthy blood volume fraction	0.1	−	Assumed
*pH*	pH-value	7.352	−	Loeppky et al. (1983)

^1^ In the same order of magnitude as in Wang et al. (1976)

^2^ The solubility of water is used to account for the solubility in blood, as well as in the alveolar epithelium and capillary endothelium, whose cytoplasm primarily consists of water (Klinke et al. 2010)

^3^ The same value as for oxygen is used, as the diffusion coefficient is determined by the molecular diameter, which is nearly identical for both gases (Klinke et al. 2010)

^4^ Physiological value for a 25-year-old 1.80 m tall woman (Klinke et al. 2010)
